# Complete Genome Sequence of *Komagataeibacter hansenii* Strain SC-3B

**DOI:** 10.1128/genomeA.00169-17

**Published:** 2017-04-13

**Authors:** Sarah Pfeffer, Richard Santos, Marcus Ebels, Darius Bordbar, R. Malcolm Brown

**Affiliations:** Molecular Biosciences, College of Natural Sciences, University of Texas at Austin, Austin, Texas, USA

## Abstract

This study reports the release of the complete nucleotide sequence of *Komagataeibacter hansenii* SC-3B, a new efficient producer of cellulose. Elucidation of the genome may provide more information to aid in understanding the genes necessary for cellulose biosynthesis.

## GENOME ANNOUNCEMENT

*Komagataeibacter hansenii* strains (formerly *Gluconacetobacter xylinus* and *Acetobacter xylinum*) have been found to be efficient producers of a pure-form cellulose synthesized through a hierarchical cell-directed self-assembly process (1 – 4). As a result of this process, the subsequent membrane formed at the air–liquid interface possesses unique properties. Its ultrafine reticulated structure, high crystallinity, great mechanical strength, high water-holding capacity, moldability during formation, and biocompatibility make it well suited for medical, industrial, and commercial applications (5 – 8). To aid in the understanding of the mechanisms needed to guide this assembly process, this study reports the release of the complete nucleotide sequence of a novel strain, *K. hansenii* SC-3B.

*K. hansenii* SC-3B was isolated from Kombucha tea (Kombucha Kamp, Beverly Hills, CA, USA), and from initial observations we determined that it is an efficient producer of bacterial cellulose. DNA was extracted and subjected to sequencing using an Illumina HiSeq 2000 PE100 system (University of Texas at Austin, ICMB Core Facility). The reads were downloaded into Geneious version 8.1.2 and assembled into contigs using Velvet version 1/2/02 (9), where it was revealed that the genome is approximately 3.64 Mb in size with a G+C content of 59.6% (10). A total of 3,792 open reading frames were predicted using Glimmer (11). Preliminary annotation data on contigs containing cellulose synthase genes were determined.

Preliminary phylogenetic analysis using 16S rRNA genes determined that this new strain is closely related to *K. hansenii* ATCC 23769. A homology comparison to the *acsABCD* operon of *K. hansenii* ATCC 23769 (GenBank accession no. AB091060) was performed and resulted in a 99.7% identity to *acsAB*, 99.4% identity to *acsC*, and 100% identity to *acsD*. Further investigations into the genome indicated that *K. hansenii* SC-3B contains a total of three separate coding regions for cellulose biosynthesis: *acsABCD*, *acsAII*, and *acsABC*. These three operons are also found in *K. hansenii* ATCC 23769. A homology comparison of the shared cellulose-synthesizing regions revealed a sequence identity of 76.6% identity to *acsAII* and 99.1% identity to *acsABC*. The *acsABCD* operon is flanked by genes coding for proteins which have been determined to be essential for proper cellulose biosynthesis to occur: *cmcAx, ccpAx*, and* bglAx* (12 – 15). These genes shared, respectively, 99.5%, 99.7%, and 98.9% sequence identities to *K. hansenii* ATCC 23769.

Further investigations into the genome of *K. hansenii* SC-3B may provide more insight into the mechanisms necessary for cellulose biosynthesis.

### Accession number(s).

This whole-genome shotgun project has been deposited in DDBJ/ENA/GenBank under the accession number MJMF00000000. The version described in this paper is the first version, MJMF01000000.
